# In silico study of HASDI (high-affinity selective DNA intercalator) as a new agent capable of highly selective recognition of the DNA sequence

**DOI:** 10.1038/s41598-023-32595-4

**Published:** 2023-04-03

**Authors:** Andrii A. Zaremba, Polina Yu. Zaremba, Svitlana D. Zahorodnia

**Affiliations:** Zabolotny Institute of Microbiology and Virology of NASU, 154 Acad. Zabolotny Str., Kyiv, 03143 Ukraine

**Keywords:** Cancer therapy, Genome informatics, Virtual drug screening, Drug discovery, Molecular biology, Oncology

## Abstract

Cancer as an acquired genetic disease is based on changes both in the genome itself and in transcription processes. Accordingly, it is at the DNA level that it makes sense to search for and design agents capable of effective and selective anticancer action. In this study, we used an iterative approach based on a molecular dynamics simulation to design a highly selective DNA-intercalating agent called HASDI. To confirm its selective affinity to DNA, we conducted two simulation experiments: HASDI in a complex with a DNA fragment of the EBNA1 gene (it targets 16 nucleotide pairs of this gene) and HASDI in a complex with a random DNA fragment of the KCNH2 gene. The molecular dynamics simulation was carried out in the GROMACS 2019 package. The binding energy was calculated by gmx_MMPBSA 1.5.2. The further analysis was performed using the built-in utilities of GROMACS, gmx_MMPBSA and also XMGRACE and Pymol 1.8. As a result, we determined that the EBNA1-50nt/HASDI complex was stable throughout the whole simulation trajectory. HASDI, due to the presence of a linker modified depending on a specific pair of nitrogenous bases, formed an average of 32 hydrogen bonds with a sequence of 16 nucleotide pairs. Phenazine rings were stably intercalated every 2 base pairs. The root-mean-square deviation of HASDI in such a complex fluctuated around the value of 6.5 Å and had no tendency to increase. The calculated value of the binding free energy was − 235.3 ± 7.77 kcal/mol. The KCNH2-50nt/HASDI complex, as an example of the intercalation of the designed structure into a random part of the human genome, maintained the stability of its position at a level comparable to the EBNA1-50nt/HASDI complex. The phenazine rings were constantly intercalated in their original positions, and the root-mean-square deviation fluctuated around one value, although it had a tendency to chaotic changes. At the same time, this complex was characterized by 17–19 hydrogen bonds, on average, and the binding free energy was − 193.47 ± 14.09 kcal/mol. Moreover, the DNA duplex had local single-nucleotide melting in the region of the 4th linker. According to a significant decrease in the number of hydrogen bonds, a decrease in energy gain, as well as a decrease in the stability of the DNA duplex characteristic of the KCNH2-50nt/HASDI complex compared to the target EBNA1-50nt/HASDI complex, the molecule we designed can be considered a potentially selective DNA polyintercalating agent capable of relatively accurate recognition of 16 base pairs.

## Introduction

The basis of the existence of all living things is the carrier of genetic information: for cellular forms of life it is DNA, for non-cellular ones, such as viruses, it is both RNA and DNA. The carrier of genetic information is the basis and reason for all long-term changes in the ontogenesis of any organism. These changes can be present from birth and characteristic of the species, like a specific growth and development of organs and tissues, or present from birth and not characteristic of the species, such as various congenital pathological conditions. Furthermore, they can be acquired in the process of ontogenesis, e.g. benign and malignant degeneration of cells.

However, the existence of a genome alone is not enough to manifest the changes encoded in it. It is necessary for the genetic information to be implemented. For DNA-containing life forms, the first stage of this process is transcription: the transfer of the sequence from a low-mobility but stable carrier to a more labile and short-lived one, i.e. to RNA. This process involves the work of various factors that collectively provide unwinding, relaxation, and reading of the DNA sequence followed by RNA synthesis. If any of these processes become difficult or impossible, the transcription process will be erroneous or even impossible and genetic information will not have any manifestations.

At the same time, most of the current homeostasis disturbances are caused by changes at the genome level. Accordingly, even at the modern level of understanding of the functioning of living things, almost all violations can be nullified by influencing the transcription of genetic information. Necessarily, such influence must be selective.

One of the methods of the selective influence is the inhibition of the DNA unwinding and relaxation machinery by stabilizing its duplex form with molecular structures capable of intercalation between nitrogenous bases^1^. This principle is already practically used now. It is the basis of the action of many anticancer drugs (daunomycin, doxorubicin, thalidomide). The latter, however, have a huge pool of various severe side effects in addition to the positive ones^2–4^. This results in the complexity of their application and, accordingly, limited effectiveness. The main cause of side effects is the low specificity of such agents. They interact with the entire DNA of the body, and only the mitotic potential of a specific tissue determines its sensitivity to cytostatics of this type.

In this work, we would like to present HASDI—the new polyintercalating agent potentially capable of highly selective recognition of the nucleotide sequence of the DNA duplex, designed by using an iterative approach based on molecular dynamics simulation. We show that the designed molecule has a higher affinity for the targeted sequence than for a randomly selected sequence of the hERG gene.

## Methods

### Preparation of input data

The 50-nucleotide sequences used in this study were obtained from GenBank and RefSeq: 5′-TGGAGGTAGTAAGACCTCCCTTTACAACCTCAGGCGAGGAATTGCCCTTG-3′ (MT164472.1 (204–253); EBNA1), 5′-AGGCGCTGCCCGAGCCGCGGGCGCTGGAGCGGCTGTCGGCGCGGTGGCAG-3′ (NM_000238.4 (28–77); KCNH2). Classic Watson–Crick duplexes based on these sequences were generated using the Avogadro molecular editor^5^.

The creation of ligand complexes with each of the DNA fragments (both at the stage of iterative development and at the stage of final research) involved the intercalation of phenazine rings between the neutral base pairs of the selected fragments (5′-TCCCTTTACAACCTCA-3′ (221–236) for EBNA1-50nt and 5′-CGGGCGCTGGAGCGGC-3′ (45–60) for KCNH2). This was done manually and included an energy minimization session using the built-in Avogadro functional (UFF force field, Steepest algorithm).

### Simulation of molecular dynamics

Molecular dynamics simulations were performed using the GROMACS 2019.6 software package^6^, the AMBER99SB force field^7^ and the TIP3P three-point water model^8^. Ligands were parameterized in the ACPYPE using GAFF^9^ as a force field and Gasteiger as a charge method. In the process of building the complete system, the DNA/ligand complex was placed in a box of triclinic form, which was filled with a clear solvent with a physiological concentration of NaCl (0.156 M), so that the total charge of the system was equal to zero. The distance between the complex and the walls of the box was kept at 30 Å. Periodic boundary conditions were used in this study. Electrostatic energy was calculated using the PME (Particle-Mesh Ewald) method. Coulomb and van der Waals interactions had a limiting distance of 1.2 nm. The energy minimization was carried out using the steepest descent algorithm to a system energy value of less than 1000 kJ/mol/nm (50,000 steps). Equilibration was carried out in two consecutive phases, each with a length of 100 ps. First, there was the equalization of the temperature (300 K), then of the pressure (1 atm). The direct simulation of molecular dynamics was carried out for 150 ns, with a time step of 2 fs.

### Calculation of the binding free energy

The gmx_MMPBSA 1.5.2 package was used to calculate the free energy using the MM/PBSA (Molecular Mechanics/Poisson–Boltzmann Surface Area) method^10^. The source of input data was the trajectory (last 90% of frames) generated by GROMACS in the process of molecular dynamics simulation of target associates. The dielectric interface was implemented using the level setting function^11^. At the same time, nonpolar solvation free energy was modeled with SASA (solvent accessible surface area)^12^. External dielectric constant was equal to 80 and internal dielectric constant was 2. The contribution of the entropy component was calculated by the IE (interaction entropy) method^13^.

### Analysis of the obtained results

The results were analyzed using standard software provided by GROMACS (trjconv, rms, hbond) and gmx_MMPBSA (gmx_MMPBSA_ana). Visualization was carried out using XMGRACE^14^ and Pymol 1.8.

### Iterative design of a selective intercalator molecule

**Figure 1:**
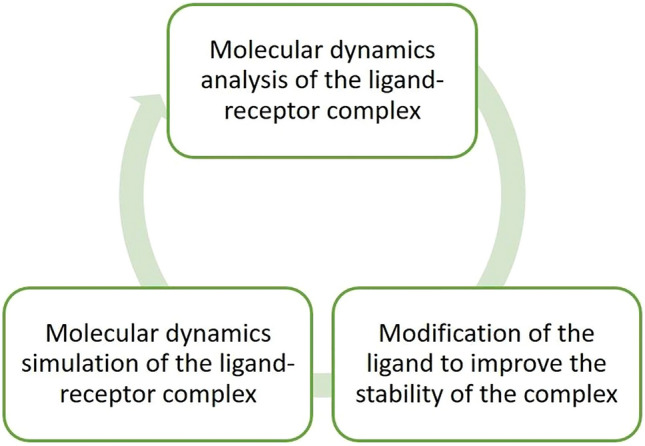
The flow chart of the iterative development process of HASDI.

Each EBNA1-50nt/ligand complex was studied by molecular dynamics simulation. After that, the linker, aliphatic in the first stages, which connects two phenazine rings, was modified in the direction of its ability to selectively recognize each of the vacant donors or acceptors of the hydrogen bond of a pair of nucleobases over which it was directly located (the major groove of the DNA duplex). Each modification was accompanied by optimization of the geometry of the complex by applying a short session of the energy minimization using the built-in Avogadro functional. The next stage was the molecular dynamics simulation of the target DNA with a new ligand. The results of the experiment were also analyzed and the cycle was repeated until, in our opinion, a sufficient level of selectivity appeared in the newly created structure (Fig. 1).

Later, the other linker with another phenazine at the end was attached to the ligand at the 9th position of one of the phenazine rings. The phenazine also underwent intercalation between the EBNA1-50nt base pairs. Thus, the pre-prototype of the recognition structure was extended to the required size for recognition of 16 base pairs. After each extension, the entire cycle of finding the optimal linker structure was repeated.

In the search process, we tried to adhere to the maximum structural similarity between different linkers, since we consider it useful for reasons of overall stabilization of the DNA/intercalator complex. The two strands of the DNA duplex, despite the possible difference in sequence, are very similar at the level of secondary structure. Accordingly, this property must also be supported by the ligand. Especially in the case of its significant size. It is also important for the relatively easy resizing of the structure and the possible subsequent process of its retargeting to other specific parts of the genome.

## Results

### Determination of the minimum number of recognized nitrogenous bases required for selective interaction

According to the fact that the human genome contains 3,099,734,149 base pairs, a structure capable of selective recognition of a certain sequence in its composition must have a size no smaller than a certain minimum. The one that, under the condition of random selection, will have a number of possible combinations of nitrogenous bases of a fragment of the same size multiplied by the number of nucleotides in its composition greater than the size of the human genome. According to the fact that each subsequent position undergoes a choice among the four possible nucleobases i.e., Adenine, Guanine, Thymine and Cytosine, the inequality will look like this:

$${4}^{{\text{m}}} *{\text{ m }} > 3{,}099{,}734{,}149$$

where m is the number of nucleotides in the DNA fragment that is recognized.

If m = 14, then the number of possible combinations is 268,435,456. Accordingly, the size of the genome, among which one such fragment can be found on average, is equal to 3,758,096,384 base pairs.

Thus, to identify a specific section of the human genome, the minimum number of recognized base pairs should be 14. In this work, we focused on recognizing 16 base pairs.

### HASDI: general description

**Figure 2:**
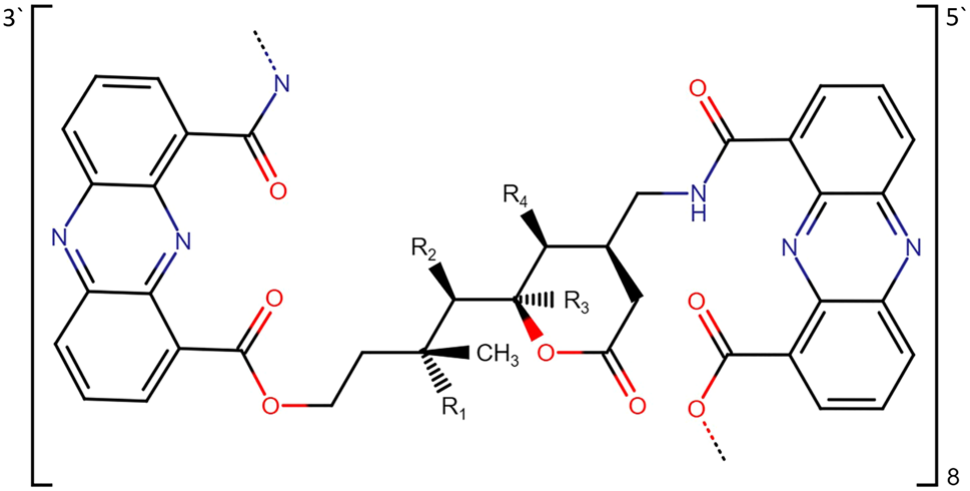
General 3D structure of HASDI. It consists of elementary blocks. Each block consists of a branched linker, where R_1_, R_2_, R_3_ and R_4_ are radicals that directly recognize the sequence of base pairs and two phenazine rings intercalating between every two nucleobase pairs. HASDI includes eight such blocks for recognition of 16 base pairs.

The high-affinity selective DNA intercalator (HASDI) created using the iterative approach consists of eight segments, each of which can recognize two base pairs (Fig. 2). Accordingly, it is able to recognize 16 pairs of nucleobases in total.

The recognition is fundamentally based on the presence of three hydrogen bond formation points from each base pair of the target sequence directed into the major groove of the DNA duplex. For the A-T pair, it is, starting with purine, acceptor (N), donor (NH), acceptor (O). For the G-C pair, it is acceptor (N), acceptor (O), donor (NH), respectively. Depending on the sequence, the pattern of spatial arrangement of hydrogen bond donors and acceptors changes. Thus forming a unique surface of a major groove, which is read by HASDI. The latter occurs through the formation of hydrogen bonds between the mentioned three polar points of the base pair and the corresponding radicals of the ligand linker.

In general, all HASDI linkers are similar, regardless of the sequence they recognize. Each is based on δ-valerolactone and 2-methylbutyl, connected by a C–C bond. To directly target each specific base pair, the linkers are modified in positions 1, 2 of the aliphatic methylbutyl chain (R_2_ and R_1_, respectively) and 5, 6 of the δ-valerolactone ring (R_4_ and R_3_, respectively). The specific functional groups among which we obtained the best results for recognizing the sequence 5′-TCCCTTTACAACCTCA-3′ (the central part of the EBNA1-50nt duplex) are presented in Table 1.

**Table 1:** Modifications of the HASDI linker aimed at recognizing different nucleic bases in the DNA duplex.

Radical	Base pair that is recognized	Substituent
R_1_	Adenine (A)	
	Guanine (G)	
	Thymine (T)	
R_2_	Adenine (A)	
	Cytosine (C)	
	Thymine (T)	
R_3_	Adenine (A)	
	Guanine (G)	
	Thymine (T)	
R_4_	Adenine (A)	
	Cytosine (C)	
	Thymine (T)	

As we mentioned in the “Methods” section, in addition to regions highly specific to the DNA sequence, HASDI is also characterized by the presence of phenazine rings that flank each linker and act as a bridge for their combination into the overall structure. Due to the low degree of polarization and the presence of a massive conjugated π-system, planar phenazine rings easily participate in hydrophobic and van der Waals interactions. The above ensures their high and non-specific affinity for DNA and leads to effective intercalation between base pairs. Thus, the phenazine chromophores in HASDI should be considered as its non-specific component, and the linker sections modified in positions R_1_, R_2_, R_3_ and R_4_ should be reviewed as a specific one. In this case, electrostatic forces can be considered as additional non-specific forces. Positively charged amino groups are used for recognition of thymine by means of R_1_ and R_4_, which, in addition to specific recognition of nitrogenous bases by means of hydrogen bonds, provide HASDI with a certain level of non-specific affinity to the negatively charged DNA backbone. It is believed that electrostatic attraction is the most important in the first stages of the interaction of a small intercalating ligand with DNA^15^.

### Molecular dynamics simulation of the EBNA1-50nt/HASDI complex

During the entire simulation period the EBNA1-50nt/HASDI complex was very stable.

Stack-interactions of all phenazine rings remained unchanged during the simulation period: each intercalating planar structure was constantly in the place of its initial placement, that is, at a distance of 2 base pairs from each other.

Selective interactions differed depending on the specific sequence (Table 2).

**Table 2:** Features of the interaction of HASDI with the sequence 5′-TCCCTTTACAACCTCA-3′ (EBNA1-50nt).

No (5′ to 3′)	Base pairs	HASDI recognition of the EBNA1-50nt fragment (5′-TCCCTTTACAACCTCA-3′)	Average number of hydrogen bonds
1	T-A C-G		6
2	C-G C-G		6
3	T-A T-A		6
4	T-A A-T		6
5	C-G A-T		6
6	A-T C-G		6
7	C-G T-A		6
8	C-G A-T		6

Hydrogen bonds between linkers and polar atoms of nucleobases are indicated by a yellow dashed line.

Each base pair can be recognized by a maximum of three hydrogen bonds. Accordingly, each linker modified in positions R_1_, R_2_, R_3_ and R_4_ is capable of forming 6 hydrogen bonds with a pair of nucleobases. This results in a total ability of HASDI to recognize a pattern of polar atoms that includes 48 points. The analysis of the molecular dynamics simulation trajectory of the HASDI complex with the sequence to which it was actually designed (5′-TCCCTTTACAACCTCA-3′) indicates the preservation of all predicted hydrogen interactions throughout the simulation period. Thus, the position of all linkers and, accordingly, associated phenazines is fixed relative to a specific base pair and to each other.

**Figure 3:**
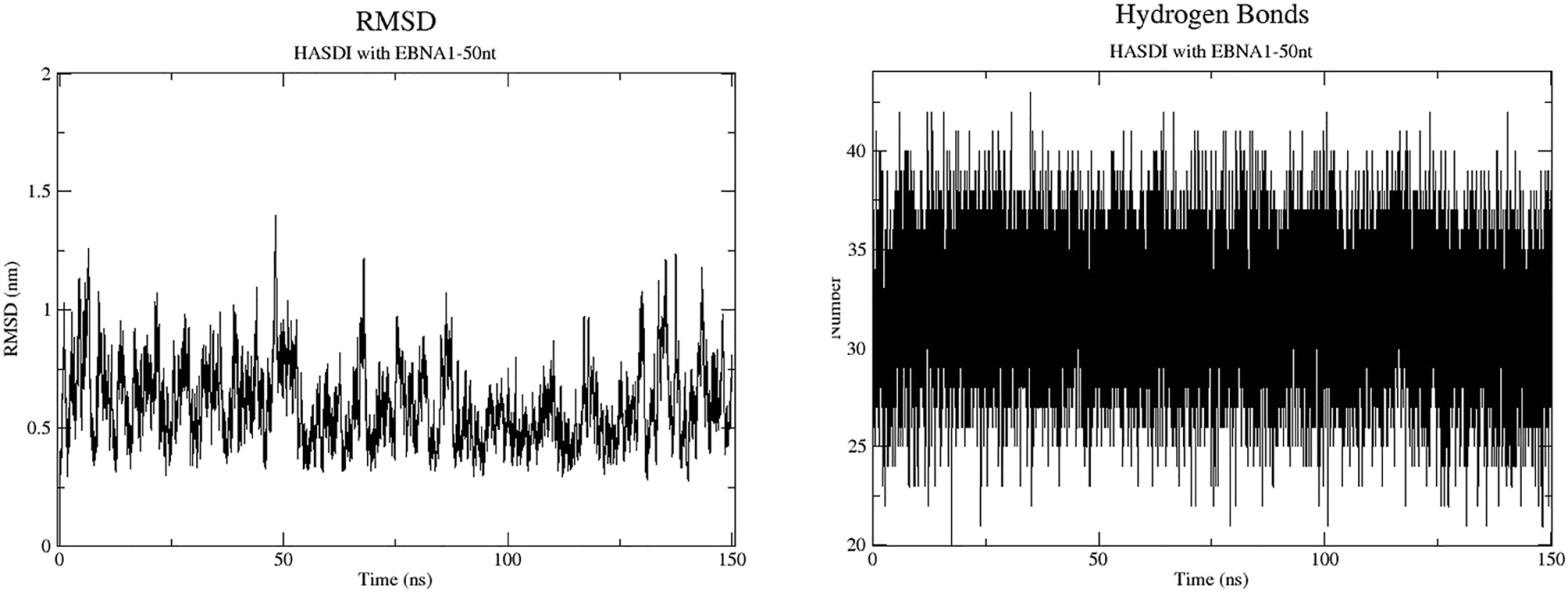
The root-mean-square deviation of HASDI in complex with EBNA1-50nt and the number of hydrogen bonds between it and the sequence 5′-TCCCTTTACAACCTCA-3′. The RMSD shows periodic undulating movements around the value of 6.5 Å without a growing tendency. The number of hydrogen bonds also does not change and fluctuates stably around the average value of 32 bonds.

However, according to the generated hbond (GROMACS) graph of the number of hydrogen bonds versus simulation time, at each specific time interval the number of hydrogen bonds between the ligand and DNA never exceeded 43 and on average fluctuated around 32 (Fig. 3). This is not equal to the 48 possible hydrogen bonds we observed in the simulation trajectory analysis and and points in favor of a dynamic interaction between HASDI and the target DNA. Namely, in one local minimum, some hydrogen bonds are more likely to be formed, while others are more likely to form in another.

The root-mean-square deviation of HASDI in the target complex fluctuated around the value of 6.5 Å with peaks up to 10 Å and minima up to 3.5 Å. This is a high value for both the overall RMSD and its range. However, it should be noted that the general trend of the root-mean-square deviation is not growing, but on the contrary is surprisingly stable. In addition, a significant range of oscillations is a consequence of general undulating motions of the RMSD value, and in the case of an estimate of the local range did not exceed 2 Å.

The free energy of HASDI binding to EBNA1-50nt estimated by gmx_MMPBSA had a deeply negative value (− 235.3 ± 7.77 kcal/mol), which also suggests a certain level of affinity of HASDI to the target DNA duplex.

### Molecular dynamics simulation of the KCNH2-50nt/HASDI complex

Among the entire fragment, HASDI was intercalated in the region of the sequence 5′-CGGGCGCTGGAGCGGC-3′, which is significantly different from the sequence 5′-TCCCTTTACAAACCTCA-3′.

Despite the mismatch of the KCNH2-50nt DNA sequence to HASDI, at the level of a non-specific component, its molecular dynamics was similar to those of EBNA1-50nt/HASDI. In particular, each planar phenazine ring was invariably located at its original intercalation site without undergoing significant changes in position.

At the same time, significant changes in the interactions characteristic of a specific component were observed. Among the entire sequence of KCNH2-50nt, in the central part of which HASDI was intercalated, there were two single-nucleotide overlaps with the sequence 5′-TCCCTTTACAAACCTCA-3′—A55 and C57, which interact with R_4_/R_3_ of the 6th linker and R_4_/R_3_ of the 7th, respectively (Table 3). In both cases, stable preservation of 3 hydrogen bonds with each pair of nucleotides was observed. The remaining base pairs of the non-target fragment were limited to an average of 1–2 stable bonds per linker not designed for their recognition. Interesting aberrations were observed in the case of the interaction of HASDI with DNA in the region of the 4th linker (R_4_/R_3_). From the beginning of the simulation, this region was as unstable as other regions of interaction of the ligand with non-target nucleobases in the major groove and formed an average of 2–3 hydrogen bonds. However, starting from the 93rd ns of the simulation, C51 left the duplex plane, which led to the stabilization of the linker and the formation of as many as 5 hydrogen bonds. Among other things, it should be noted that the hydroxyl group R_4_ of the first linker also acted as a donor in the formation of a stable intramolecular bond with the oxygen of the amide group.

**Table 3:** Features of the interaction of HASDI with the sequence 5′-CGGGCGCTGGAGCGGC-3′ (KCNH2-50nt).

No (5′ to 3′)	Base pairs	Interaction of HASDI with KCNH2-50nt fragment (5′-CGGGCGCTGGAGCGGC-3′)	Average number of hydrogen bonds	Notes
1	C-G G-C		2	An intramolecular hydrogen bond is present
2	G-C G-C		1	–
3	C-G G-C		1	–
4	C-G T-A		(5)	Cytosine leaves the plane of DNA after 96 ns of simulation
5	G-C G-C		2	–
6	A-T G-C		3	The A-T pair is the first overlap between EBNA1-50nt and KCNH2-50nt
7	C-G G-C		4	The C-G pair is the second overlap between EBNA1-50 nt and KCNH2-50 nt
8	G-C C-G		2	–

Hydrogen bonds between the linker regions of the ligand and the polar atoms of the nucleobases are indicated by a yellow dashed line.

**Figure 4:**
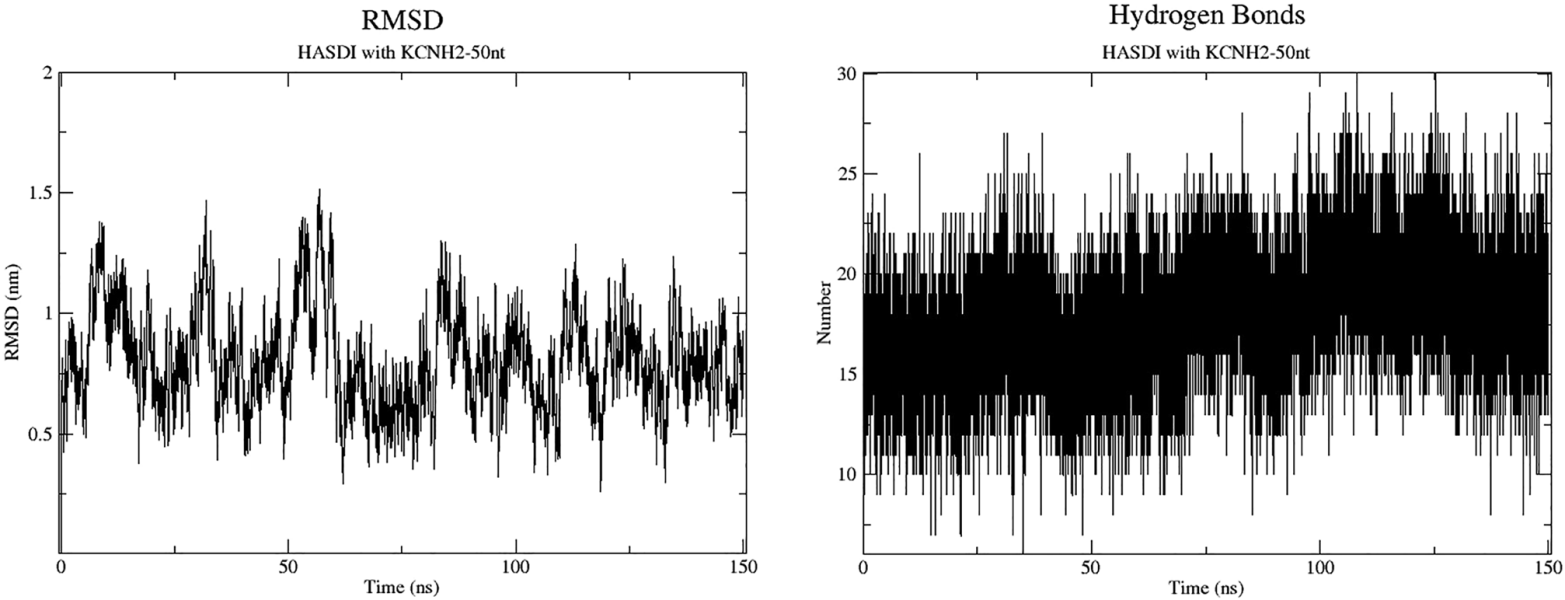
The root-mean-square deviation of HASDI in complex with KCNH2-50nt and the number of hydrogen bonds between it and the sequence 5′-CGGGCGCTGGAGCGGC-3. The RMSD shows a significant level of amplitude of the oscillations, which become periodic towards the end of the simulation. During this period, their range varies within 6 Å. The number of hydrogen bonds also fluctuates with the simulation time and at first increases to 19 and then tends to decrease. Their average number ranges from 17 to 19.

Thus, in the process of molecular dynamics simulation trajectory analysis, we observed an average of about 17–20 hydrogen bonds between the specific HASDI component and KCNH2-50nt. These observations, in this case, are confirmed by the more detailed view of the number of hydrogen interactions over time generated by GROMACS (Fig. 4). The calculated number of hydrogen bonds by hbond (GROMACS) between HASDI and KCNH2-50nt changed dynamically over the simulation time. At the beginning, their average number was about 16. After that until 110 ns of the simulation, the number increased and reached an average of 19 at the peak. Later and until the end of the simulation it decreased again to 17. The maximum peak number of 25 hydrogen interactions was observed at 108 ns. That is a much lower value even than the average number of hydrogen bonds characteristic of the EBNA1-50nt/HASDI complex.

The RMSD of HASDI in the complex with KCNH2-50nt fluctuated significantly during the molecular dynamics simulation. From the beginning, fluctuations of the RMSD from 4.5 Å up to 14.5 Å can be observed, which gives a total range of about 10 Å. This is even greater number than for HASDI in complex with EBNA1-50nt and is a consequence of pronounced undulating movements of the RMSD value. As well as the local range of the root-mean-square deviation of 4 Å. However, the above is more characteristic of the first part of the simulation. Starting from 85 ns, there is an increase in the degree of periodicity of undulating movements of the RMSD, as well as reducing their range to 6 Å. At the same time, the local scope decreased to 3 Å.

The calculated free energy of complex formation of HASDI with KCNH2-50nt similarly to HASDI/EBNA1-50nt is deeply in negative values and equals to − 193.47 ± 14.09 kcal/mol. This indicates in favor of the preservation of a certain level of affinity in HASDI to non-specific regions of DNA, although lower than to EBNA1-50nt.

## Discussion

Attempts to increase the selectivity of the interaction of a small non-covalent ligand with DNA, which acts as a receptor, are one of the important and widespread areas of drug design that affect the functioning of the latter^16^. Some progress has already been made in this direction. For example, Otsuki et al. developed a PI polyamide targeting interaction with the minor groove of DNA encoding human TGF-β1^17^. The researchers showed a significant effect of the created polyamide on TGF-β1 expression in models of chronic nephropathy and unilateral urethral obstruction (marmoset). In particular, an immunohistochemical study revealed a complete inhibition of TGF-β1 staining in the medulla of the kidneys of marmosets. In subsequent studies, the inhibitory effect of PI polyamide on the synthesis of TGF-β1 in diabetic nephropathy^18^ and in liver and lung cancer cells^19^ was confirmed. Thus, the researchers proved the selectivity and efficiency of the structure they designed. Another group of scientists in the course of their research designed a PI polyamide specific for a tandemly repeated telomeric region with a length of 24 bp^20^. There are other studies where a designed agent selectively interacts with the minor groove of DNA. On average, they are limited by the presence of increased affinity in the studied sequence to A-T or G-C enriched sections of DNA^21–23^.

There are also studies where agents capable of selective binding to the major groove are being designed and investigated. In particular, Willis and Arya demonstrated the potential of the neomycin-Hoechst 33258-pyrene conjugate to interact with both major and minor grooves^24^. This construct can also be stabilized by intercalation between base pairs. The use of fluorescence titrations proved the ability of this conjugate to selectively recognize up to 9 A-T base pairs.

Another method of DNA recognition is the above-mentioned direct intercalation between its base pairs. Although DNA-intercalating substances are small compared to the rest of the compounds, they also have a certain level of selectivity, mostly to G-C base pairs^25–27^. In this case, the bis-intercalator MLN944 (XR5944), which is currently at the stage of clinical studies, deserves some special attention^28^. Its linker, which connects two phenazine carboxamides, is positively charged and is located in the major groove of DNA. It shows the greatest affinity to sequences of the d(ATGCAT)_2_ type due to the location of its diamine linker in the major groove and the interaction of the latter with G and C. Thus, this molecular construct, despite its relatively simple structure, selectively interacts with DNA. This process involves intercalation between base pairs to acquire non-specific affinity and hydrogen bonds to acquire sequence specificity.

Summarizing the current state of development of DNA sequence-selective agents, it can be seen that only a few among a significant number of studies have reached the level of the ability to recognize a specific gene. So far, these discoveries are also limited to individual studies. Most other works stop at the development of a small molecule capable of recognizing some short consensus sequence.

The above studies are focused on proving the selectivity and efficiency of previously obtained compounds by various methods. At a more modern level, the first stages of the selection and design of potentially active structures, as well as their initial testing, are increasingly conducted in silico. For example, El-Adl et al. proved that their [1,2,4]Triazolo[4,3-c]quinazoline and bis([1,2,4]triazolo)[4,3-a:4′,3′-c] quinazoline derived DNA intercalators created by structure-based design have increased activity against cancer cell cultures compared to doxorubicin^29^. At the same time, their high activity is well consistent with the calculated component of their research. Another variant of the application of simulation studies concerns the determination of the mechanism of interaction of drug candidates with their target factors. For example, Tumbi et al. determined that the active derivatives 5F-203 and 5-aminoflavone have specificity for minor or major groove as well as for DNA sequence, using molecular docking, molecular dynamics simulation, MM-PBSA and MESP^30^. In particular, 5F showed affinity only to the minor groove of the studied DNA sequences. At the same time, AMF is stable in the minor groove of AT-rich DNA and in the major groove of GC-rich DNA. Another interesting study is described in a recent publication by Maganti and Bhattacharyya^31^. A significant part of the potential of calculations is used there to study the molecular mechanisms of interaction at the atomic level. To study the specificity of daunomycin (a classic DNA intercalator) to various DNA sequences, the scientists applied both classical molecular dynamics simulation methods and MM‑PB(GB)SA, as well as DFT analysis adapted by them for such calculations. A significant amount of various virtual experiments was conducted, allowing the researchers to state that daunomycin has the highest affinity for TC/GA rich sequences. Previously, this was also proven in vitro^32^. In addition, the researchers quite reasonably claim that the approach they used to evaluate the mechanism of interaction of DNA with small intercalating ligands can also be used to improve the efficiency of the latter.

Current research is limited in the direction of selective DNA recognition by small molecules, and in silico studies have high potential for the development of new DNA recognition agents. Based on this, there is sense and perspective in the development by computational methods of structures capable of selective recognition of a specific, possibly irregular sequence with a length sufficient to distinguish it from the rest of the genome.

In this study, we focused on the design of a molecular construct capable of accurately recognizing the sequence it targets. It is assumed that this design should be of sufficient size to recognize the minimum number of base pairs required to identify a specific sequence in the human genome. Also it should have the potential for relatively simple retargeting to other parts of the genome.

According to our calculations, the size of such a sequence for a human with its more than 3 billion base pairs should be at least 14 bp. To better identify a specific sequence, we chose to target 16 bp, which allows to recognize such a fragment among more than 68 billion bp.

The structure designed by us, using an iterative approach based on molecular dynamics simulation, was named HASDI from "high-affinity selective DNA intercalator". It is based on phenazine rings, which are characterized by low polarity and conjugated π-system. This in total allows phenazine to participate in the formation of interplanar stacking with other similar structures, for example, nitrogenous bases in the composition of nucleic acids^33^. In this case, the phenazine ring acts as an effective intercalating agent, which itself does not have significant specificity for the DNA duplex sequence. In this way, we ensure general and non-selective affinity of HASDI for DNA. Phenazine rings are combined linkers based on δ-valerolactone and 2-methylbutyl connected by a C–C bond. The latter, in turn, are modified in positions 1, 2 of the aliphatic methylbutyl chain (R_2_ and R_1_, respectively) and in positions 5, 6 of the δ-valerolactone ring (R_4_ and R_3_, respectively) depending on the sequence. Substituents in these positions, together with the scaffold, are located within the DNA major groove and each form hydrogen bonds with its nitrogenous base. Substituents R_4_ and R_3_, like R_2_ and R_1_, lie in the same plane and are directed in opposite directions. Each pair of substituents allows the formation of 3 hydrogen bonds to recognize one base pair, the donors and acceptors of which are directed into the major groove. Thus, one linker recognizes only two base pairs (through 6 hydrogen bonds), which are flanked on both sides by phenazine rings intercalated in DNA. The next two base pairs are recognized in a similar way. Then one of the phenazine rings acts as a kind of bridge connecting neighboring linkers.

More generally, the structure described above is modular. Each module is capable of recognizing two base pairs in the target sequence. This recognition is realized due to the formation of hydrogen bonds between the linker sites of HASDI and the donor–acceptor surface of the DNA major groove. Such a structure can be extended until it reaches the required length. However, it should be remembered that the physico-chemical and pharmacokinetic parameters deteriorate with the increase in the size of the molecule.

In this work, we study HASDI targeted at a short sequence within the EBNA1 gene of the Epstein-Barr virus (5′-TCCCTTTACAACCTCA-3′).

In the complex with the DNA fragment of 50 bp, which contains the target sequence, the designed structure stably kept its position during the entire period of molecular dynamics simulation (150 ns). The analysis of the simulation trajectory allows us to assert that the interactions, which we previously divided into specific (hydrogen bonds) and non-specific (hydrophobic, van der Waals and ionic interactions), are the basis of this constant preservation of the position. In particular, all phenazine fragments, despite the presence of some degree of freedom in the direction perpendicular to the duplex axis, on average stably intercalated in the same positions, and therefore preserved the entire spectrum of those nonspecific interactions, as at the beginning of the experiment. This indicates the presence of a significant degree of affinity of HASDI to DNA itself. The linker regions modified according to the DNA sequence, despite the presence of a much larger number of rotational bonds, also constantly kept their position—each clearly above its base pair. This is a consequence of the large number of hydrogen bonds present during the interaction of each individual linker with the recognized base pair. Despite the apparent preservation of all 48 hydrogen bonds, which we observed during the analysis of the simulation trajectory, a more detailed evaluation allowed us to determine that their number fluctuated around the value of 32 bonds, on average. This means that there are 4 hydrogen bonds for each base pair recognized by one linker instead of 6. Combining this with the trajectory data, it can be argued that in this case the pattern of hydrogen interactions is constantly and dynamically changing, keeping on average 4 hydrogen bonds per base pair.

The root-mean-square deviation of HASDI in the complex with EBNA1-50nt, despite its high value, shows undulating movements around one value (6.5 Å) without a tendency to increase. Together with abovementioned this may indicate in favor of the high level of affinity of HASDI to the 5′-TCCCTTTACAACCTCA-3′ DNA sequence.

To understand the contribution of the selective HASDI component, we created its complex with a random fragment of the human KCNH2 gene, also 50 nt long, and intercalated it for direct interaction with the sequence 5′-CGGGCGCTGGAGCGGC-3′. Thus, we simulated binding to a random part of the human genome.

The molecular dynamics trajectory of such complex was much less homogeneous compared to the original one. Despite the fact that the nonselective interactions provided by the intercalation of phenazine rings are stable (which is expected), the pattern of hydrogen bonds has undergone both qualitative and quantitative changes. In particular, stable selective interactions were observed only in cases where there was a random one-nucleotide coincidence of the original target sequence with a randomly selected one (A55 and C57). In the remaining cases, the number of hydrogen bonds ranged from 1 to 4. According to the calculation of their total number during the trajectory analysis, it was equal to 17–20. This is consistent with their average number calculated using hbond. There, the largest number of hydrogen bonds was observed in the period of 110 ns (19 bonds on average). After that, it was decreasing to the end of the simulation. Thus, in terms of the number of directed selective interactions, the KCNH2-50nt/HASDI complex significantly loses to the EBNA1-50nt/HASDI complex.

In addition, the KCNH2-50nt duplex itself underwent conformational rearrangements: the cytidine, which was supposed to interact with the R_4_ of the fourth HASDI linker, left the duplex in the second part of the simulation. Thus a local melting of the DNA double helix was formed.

The changes, although much less impressive, are also visible in the graph of the change in RMSD over time. The initial 85 ns simulations are characterized by a significant range of undulating oscillations of the root-mean-square deviation, which is bigger compared to the original complex. However, in further oscillations approach the values characteristic of HASDI in the complex with EBNA1-50nt.

The difference between the EBNA1-50nt/HASDI and KCNH2-50nt/HASDI complexes is also evident at the level of the binding free energy calculation. In particular, the first is characterized by the complexation energy of − 235.3 ± 7.77 kcal/mol, and for the second it is − 193.47 ± 14.09 kcal/mol. Due to the limitations of the method, it makes no sense to take into account the absolute values of the energy component of complexation. However, it makes sense to take into consideration their relative ratio. In particular, the binding free energy is obviously higher in the case of HASDI interaction with the sequence 5′-TCCCTTTACAACCTCA-3′ (EBNA1-50nt), which once again confirms its higher affinity to the target DNA sequence compared to a random sequence. However, the fact that the difference between the calculated free energy values does not exceed 20% should also be taken into account. This, in turn, can be considered as a sign of the presence of a significant non-specific component in the interaction of HASDI with DNA.

Thus, it can be argued that the stability of the position of HASDI in a complex with DNA largely depends on the non-selective component of its structure. That is provided by the phenazine rings, which effectively intercalate into the duplex in the case of both the target and the non-target sequence for HASDI. However, the selective component, which is represented by a branched linker capable of hydrogen interactions, still has a significant impact on the formation of a full-fledged interaction with the target sequence. Moreover, significant enough to ensure a decrease in the average number of hydrogen bonds from 32 to 17–19, lead to local melting of the DNA duplex and a drop in the binding free energy by ~ 35 kcal/mol when interacting with a non-target sequence.

Summing up, we believe that we managed to create a modular construct with a high potential for selective recognition of a certain DNA sequence (in this study, 5′-TCCCTTTACAACCTCA-3′). At the current level, this potential is not fully realized due to the significant contribution of the non-selective component to the HASDI/DNA interaction. In further in silico research, we plan to reduce the contribution of hydrophobic and van der Waals interactions by modifying or replacing phenazine chromophores while preserving and possibly optimizing the selective HASDI component. In case of success of such studies, we are considering the possibility of in vitro testing of HASDI and its derivatives for specificity and effectiveness in controlling the functioning and implementation of a certain specific DNA sequence using the example of viral or cancer model systems. A success at this level would significantly validate our research and draw the world interest to it. This will be useful in attracting the larger community of scientists to the development of HASDI locally and attention to the design of new anticancer drugs in general.

## Conclusion

Our designed structure HASDI in this study was targeted at a fragment of the Epstein-Barr virus EBNA1 gene with a length of 50 nucleotides. In this complex, the ligand recognized each pair of nucleobases of the DNA duplex by forming at least 4 hydrogen bonds. The associate itself was characterized by the stable preservation of the RMSD and the calculated complexation energy of − 235.3 ± 7.77 kcal/mol. The complex with a random non-target DNA sequence (human KCNH2, also 50 nucleotides), despite comparable values of the RMSD, demonstrated the loss of almost half of the hydrogen bonds of the HASDI linker regions intended for specific recognition of the DNA sequence. It was also characterized by induction of single-nucleotide melting of the DNA duplex and the energy of complex formation of − 193.47 ± 14.09 kcal/mol. These data suggest that HASDI has a higher affinity to the target sequence. Accordingly, we believe that HASDI can be considered as a potentially highly selective polyintercalating agent with significant affinity for DNA.

In addition, we believe that the obtained structure has a high potential for further research in the direction of increasing its selectivity and prospects for the development of highly effective drugs based on it. At the current level of development of this concept, we see the need for a significant pool of additional in silico studies aimed primarily at reducing the non-selective interaction with DNA. Further in vitro and in vivo studies are an obvious stage in the development of HASDI as a promising drug candidate.

## Data Availability

The authors confirm that the datasets used in the current study are available in the GenBank and RefSeq repositories, MT164472.1 and NM_000238.4 respectively.
